# Ubiquinone binding site of yeast NADH dehydrogenase revealed by structures binding novel competitive- and mixed-type inhibitors

**DOI:** 10.1038/s41598-018-20775-6

**Published:** 2018-02-05

**Authors:** Tetsuo Yamashita, Daniel Ken Inaoka, Tomoo Shiba, Takumi Oohashi, So Iwata, Takao Yagi, Hiroaki Kosaka, Hideto Miyoshi, Shigeharu Harada, Kiyoshi Kita, Katsuya Hirano

**Affiliations:** 1 Department of Cardiovascular Physiology, Faculty of Medicine, Kagawa University, Kita-gun, Kagawa, 761-0793 Japan; 2 Department of Biomedical Chemistry, Graduate School of Medicine, The University of Tokyo, Tokyo, 113-0033 Japan; 3 School of Tropical Medicine and Global Health, Nagasaki University, 1-12-4, Sakamoto, Nagasaki, 852-8523 Japan; 4 Department of Applied Biology, Graduate School of Science and Technology, Kyoto Institute of Technology, Kyoto, 606-8585 Japan; 5 Division of Molecular Biosciences, Membrane Protein Crystallography Group, Imperial College, London, SW7 2AZ UK; 6 Membrane Protein Laboratory, Diamond Light Source, Harwell Science and Innovation Campus, Chilton, Didcot, Oxfordshire OX11 0DE UK; 7 Japan Science and Technology Agency, Exploratory Research for Advanced Technology, Human Receptor Crystallography Project, Yoshida-Konoe-cho, Sakyo-ku, Kyoto, 606-8501 Japan; 8 Department of Cell Biology, Graduate School of Medicine, Kyoto University, Yoshida-Konoe-cho, Sakyo-Ku, Kyoto, 606-8501 Japan; 9 Systems and Structural Biology Centre, RIKEN, 1-7-22 Suehiro-cho Tsurumi-ku, Yokohama, Kanagawa 230-0045 Japan; 10 Department of Molecular and Experimental Medicine, The Scripps Research Institute, La Jolla, California, 92037 USA; 11 Division of Applied Life Sciences, Graduate School of Agriculture, Kyoto University, Sakyo-ku, Kyoto, 606-8502 Japan; 12 Present Address: Osaka Jikei College, 1-2-8 Miyahara, Yodogawa-Ku, Osaka, 532-0003 Japan

## Abstract

Yeast Ndi1 is a monotopic alternative NADH dehydrogenase. Its crystal structure in complex with the electron acceptor, ubiquinone, has been determined. However, there has been controversy regarding the ubiquinone binding site. To address these points, we identified the first competitive inhibitor of Ndi1, stigmatellin, along with new mixed-type inhibitors, AC0-12 and myxothiazol, and thereby determined the crystal structures of Ndi1 in complexes with the inhibitors. Two separate binding sites of stigmatellin, STG-1 and STG-2, were observed. The electron density at STG-1, located at the vicinity of the FAD cofactor, further demonstrated two binding modes: STG-1a and STG-1b. AC0-12 and myxothiazol are also located at the vicinity of FAD. The comparison of the binding modes among stigmatellin at STG-1, AC0-12, and myxothiazol revealed a unique position for the aliphatic tail of stigmatellin at STG-1a. Mutations of amino acid residues that interact with this aliphatic tail at STG-1a reduced the affinity of Ndi1 for ubiquinone. In conclusion, the position of the aliphatic tail of stigmatellin at STG-1a provides a structural basis for its competitive inhibition of Ndi1. The inherent binding site of ubiquinone is suggested to overlap with STG-1a that is distinct from the binding site for NADH.

## Introduction

A monotopic alternative NADH dehydrogenase (Type II NADH dehydrogenase: NDH-2) catalyses the electron transfer from NADH to quinone via FAD or FMN without a proton-pumping activity, and functions as an initial enzyme, either in addition to or as an alternative to proton-pumping NADH dehydrogenase (complex I) in the respiratory chain of bacteria, archaea, and fungal and plant mitochondria^1–3^. NDH-2 has been attracting based on its potential medical applications. A series of studies has suggested that Ndi1, one of three NDH-2s from *Saccharomyces cerevisiae*, would be useful in replacement therapy for mitochondrial diseases caused by complex I dysfunction^4–7^. Apoptosis-inducing factor (AIF) and its family member protein AIF-M2 are mammalian members of NDH-2 family^8,9^. Although they exert NADH-ubiquinone oxidoreductase activity under certain conditions^8^, their functional role as a NADH-ubiquinone oxidoreductase in the respiratory chain is dispensable in mammals, as complex I plays a major role. Therefore, the microorganism NDH-2 has emerged as a potential therapeutic target for drugs targeting human pathogenic bacteria^10–13^ and parasites^14–16^. The quinone binding site of NDH-2 is thought to be an ideal target for chemotherapeutic agents because several compounds showing highly selective toxicity for parasites have been shown to target the quinone binding of respiratory enzymes^17–19^. Atovaquone, which has a similar structure to ubiquinone, is used as a therapeutic agent for *Plasmodium falciparum* malaria. It inhibits the respiration of malaria parasites by inhibiting the binding of ubiquinone to complex III^20^. The biochemical and structural basis for the function of NDH-2 has been intensively studied for these purposes in recent years^10–12,14–16^.

We reported that the enzymatic reaction of yeast Ndi1 has a ping-pong mechanism, as is the case with NDH-2s from *Mycobacterium tuberculosis* and *Yarrowia lipolytica*^21–23^. In this reaction mechanism, two substrates—NADH and ubiquinone—bind to the enzyme sequentially, but not at the same time. However, our previous study could not determine whether the reaction occurred via a one-site or two-site ping-pong mechanism. Recent studies that investigated the structure and biochemistry of NDH-2s proposed a non-classical two-site ping-pong mechanism in *M. tuberculosis* NDH-2^24^ and a ternary complex mechanism in yeast Ndi1^25^ and NDH-2 from *Staphylococcus aureus*^26^. NDH-2 from *Caldalkalibacillus thermarum* follows either a two-site ping-pong mechanism or a ternary complex mechanism depending on the substrate that is used^27^. NADPH favoured a two-site ping-pong mechanism, while NADH favoured a ternary complex mechanism^27^. In a ternary complex mechanism, two substrates—NADH and ubiquinone—simultaneously bind to the enzyme. Thus, these two reaction mechanisms require separate binding sites for NADH and ubiquinone.

**Figure 1:**
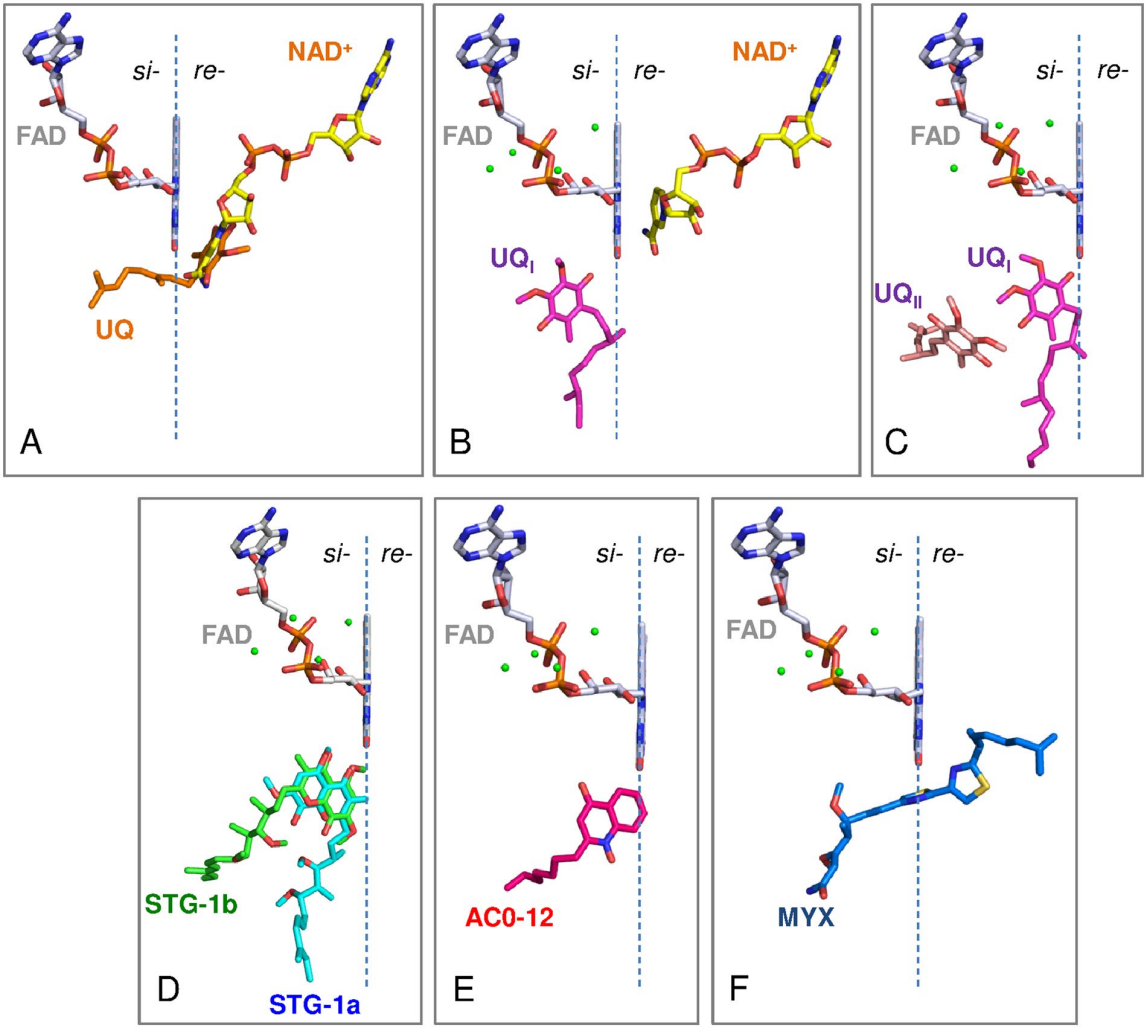
The arrangement of inhibitors and substrates in relation to the *si*- and *re*-face of the FAD cofactor. The *re*-face and *si*-face (membrane side) of the isoalloxazine ring of FAD are separated by the dashed line. The substrate molecules, NAD^+^, UQ, UQ_I_, and UQ_II_ are represented by yellow, orange, magenta, and pink sticks, respectively. (**A**) The binding model of NAD^+^ and ubiquinone that was proposed in our previous report^28^. The structures of the Ndi1-NAD^+^ (PDB code: 4GAP) and Ndi1-ubiquinone (PDB code: 4GAV) complexes are superposed to adapt this model. (**B** and **C**) The binding model of NAD^+^ and ubiquinone proposed by Feng *et al*.^29^. The structures of Ndi1-NAD^+^-ubiquinone (PDB code: 4G73; b) and Ndi1-ubiquinone (PDB code: 4G74; **C**) complexes are superposed to adapt this model. (**D**–**F**) The inhibitors, stigmatellins (STG-1a and -1b, **D**), AC0-12 (**E**) and myxothiazol (MYX, **F**), are represented by as aqua, green, hot pink and blue sticks, respectively.

We previously determined the crystal structure of Ndi1 in complex with either NAD^+^ or ubiquinone and demonstrated that the binding sites of both substrates overlap with each other and that the bound ubiquinone penetrates the plane of the isoalloxazine ring of FAD (Fig. 1A)^28^; these observations support the one-site ping-pong mechanism. In contrast, the structure of Ndi1 reported by Feng *et al*. proposed the presence of two ubiquinone binding sites (UQ_I_ and UQ_II_), both of which were located on the opposite side of the NADH binding site with respect to the plane of the isoalloxazine ring of FAD (Fig. 1B,C)^29^. This structural model is consistent with either a two-site ping-pong mechanism or a ternary complex mechanism. Thus, in order to understand the enzymatic mechanism of Ndi1, it is necessary to elucidate the reason for the inconsistency in the reported binding site of ubiquinone. One insightful strategy that could solve this problem would be to elucidate the crystal structures of Ndi1 in complex with a competitive inhibitor and to identify the site responsible for competitive inhibition, because the site contributing to the binding of a competitive inhibitor might also be involved in the binding of substrate ubiquinone. However, while many compounds, including phenothiazines and quinolones, are known to be inhibitors of NDH-2, no competitive inhibitor has been identified for NDH-2^10,14,15,21,30^.

In the present study, we screened the compounds that are known to inhibit quinone binding in several respiratory enzymes, including NDH-2, and thereby discovered the first competitive inhibitor of Ndi1, stigmatellin. Furthermore, AC0-12 and myxothiazol were newly identified as mixed-type inhibitors of Ndi1. The comparison of the crystal structures of Ndi1 in complexes with stigmatellin, AC0-12, and myxothiazol, revealed a unique binding site for stigmatellin that was distinct from the binding site for NADH. Mutations of amino acid residues that are involved in specific binding to stigmatellin reduced the affinity of Ndi1 for ubiquinone. As a result, we propose that the binding site of stigmatellin may be the binding site of the substrate ubiquinone.

## Results

### The identification of stigmatellin as a competitive inhibitor of Ndi1

**Figure 2:**
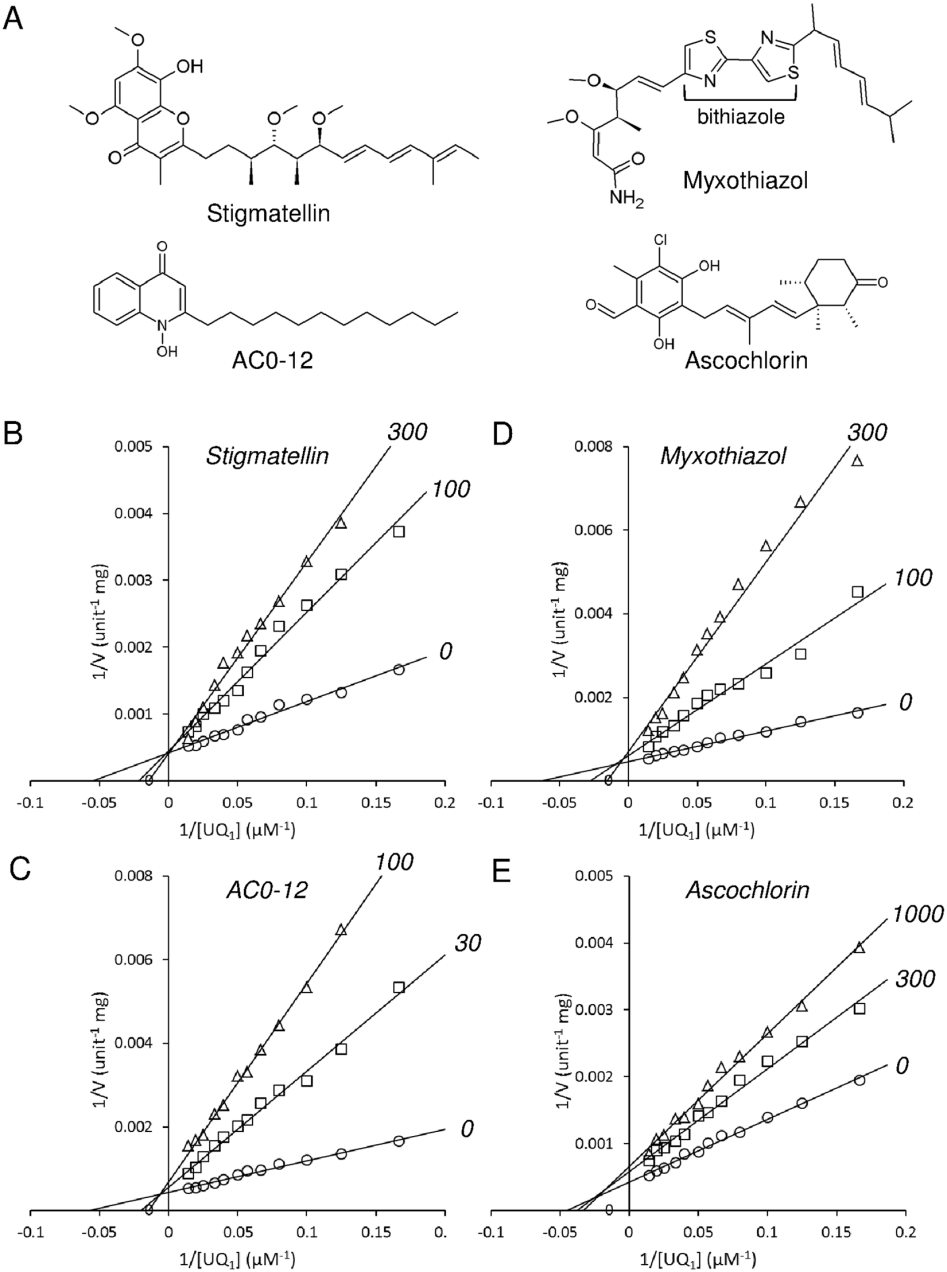
The inhibitory effects of stigmatellin, AC0-12, myxothiazol and ascochlorin on the NADH-ubiquinone oxidoreductase activity of the recombinant Ndi1. (**A**) The chemical structures of the inhibitors. (**B**–**E**) Lineweaver-Burk plots for the NADH-ubiquinone oxidoreductase activity obtained with various concentrations of ubiquinone-1 and 100 μM NADH, in the presence of stigmatellin, AC0-12, myxothiazol, and ascochlorin at the indicated concentrations in nM. The data are from the triplicate measurements of one representative assay. Three independent assays gave similar results.

The compounds that have been shown to inhibit quinone binding in several respiratory enzymes (including complex I, II and III, cyanide-insensitive alternative oxidase, and NDH-2) and their derivative compounds were screened to find novel competitive inhibitors for yeast Ndi1. First, the compounds that inhibited the NADH-ubiquinone-1 oxidoreductase activity of Ndi1 by >70% at a concentration of 10 μM were selected. Second, the compounds for which the IC_50_ values for the inhibition of the Ndi1 activity were <1 μM were selected, and the mode of ubiquinone inhibition was investigated for all of the selected compounds. Four inhibitors, including a quinolone derivative, 1-hydroxy-2-dodecyl-4(1 H) quinolone (AC0-12), and compounds with carbon skeletons that differed from quinolone, stigmatellin, myxothiazol, and ascochlorin (Fig. 2A), were newly found (Table 1). Notably, stigmatellin (IC_50_ = 107 ± 21 nM) and AC0-12 (IC_50_ = 115 ± 18 nM) exhibited higher potency than 1-hydroxy-2-undecyl-4(1 H) quinolone (AC0-11), the most potent inhibitor of Ndi1 (IC_50_ = 200 nM) ever identified^23^.

**Table 1:** Inhibitory effects of inhibitors on NADH-ubiquinone-1 oxidoreductase activity of the purified Ndi1.

Compound	IC_50_^a^ (nM)	Inhibition mode^b^	*K*_i_^*c*^ (nM)	α^c^ (α > 0)
Stigmatellin	107 ± 21.0	competitive	93.9 ± 30.4	~6.33×10^20^
AC0-12	115 ± 18.0	mixed	14.0 ± 1.9	13.0±11.7
Myxothiazol	308 ± 22.9	mixed	48.8 ± 5.2	70.2±192
Ascochlorin	955 ± 139	mixed	728 ± 191	2.64±1.87

^a^Each value represents the mean ± S.E.M of three independent experiments.

^b^Inhibition modes for ubiquinone-1 were determined by the intersection point in Lineweaver-Burk plots (Fig. 2B–E).

^c^The values for the inhibition constant *K*_i_ and α were determined by fitting the experimental data, which are the same as the Lineweaver-Burk plots, to non-linear regression of mixed type inhibition using the GraphPad Prism 6 software program. Each value represents the mean ± S.D.

A Lineweaver-Burk plot analyses (Fig. 2B–E) indicated that stigmatellin is a competitive inhibitor whereas the others are mixed-type (Table 1). This was further corroborated by the *K*_i_ and α values obtained by fitting the data to the equation of mixed model inhibition in the Prism6 software program (Table 1). The product α*K*_i_ provides a dissociation constant for ternary enzyme-substrate-inhibitor complex^31^. The α value of stigmatellin was estimated to be close to infinity, which indicated the competitive nature of the inhibition of the Ndi1 oxidoreductase activity by stigmatellin. On the other hand, the finite and greater-than-one α values of other compounds were consistent with their inhibition of the Ndi1 oxidoreductase activity being of the mixed type.

### Structure determination

Ndi1-stigmatellin, -AC0-12 and -myxothiazol complexes were successfully crystallized by cocrystallization, but diffraction-quality crystals of the Ndi1-ascochlorin complex could not be obtained. The dimer structures of the Ndi1-stigmatellin, -AC0-12 and -myxothiazol complexes were determined at resolutions of 1.85 Å, 3.4 Å, and 3.2 Å, respectively, by molecular replacement using the inhibitor-free Ndi1 structure (PDB code: 4G9K) as a template (Supplementary Table S1). In the crystals of the Ndi1-AC0-12 and -myxothiazol complexes there is one dimer in each asymmetric unit, whereas protomers of the Ndi1-stigmatellin dimer are related by a crystallographic two-fold axis. There are no significant differences between the structures of the complexes, as indicated by root-mean-square deviations of 0.58–0.76 Å, which were calculated for 454–462 superimposed Cα positions.

### Two stigmatellin binding sites

**Figure 3:**
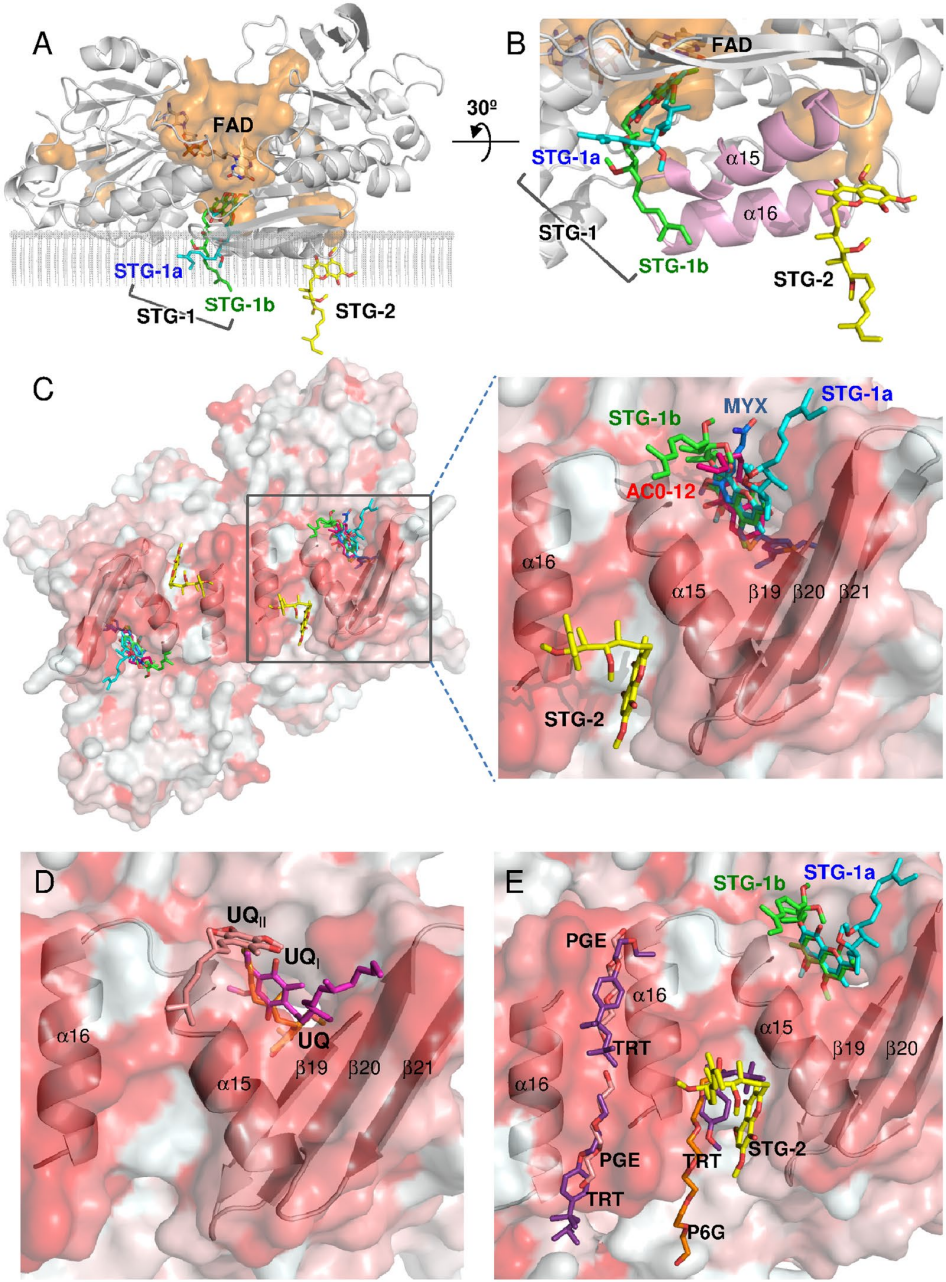
The binding sites of the inhibitors in the crystal structures of the Ndi1-inhibitor complexes. (**A**) An overall view of the Ndi1 monomer in complex with the FAD cofactor and stigmatellin with the backbone of Ndi1 shown in transparent grey. The cavities in the orange surface are shown in parallel to the cross-section of the membrane. Three different modes of stigmatellin binding are shown: STG-1a (aqua), STG-1b (green), and STG-2 (yellow). (**B**) A close-up view of stigmatellin binding at three sites in relation to the binding site of FAD and the groove formed by the α15 and α16 helixes of Ndi1 (pink). The view in A is rotated by 30° in B. (**C**–**E**) The view of one dimeric structure of Ndi1 viewed from the membrane-anchor side. The β19-21 sheets and the α15 and α16 helixes in Ndi1 forming the cavities for inhibitors are represented by a cartoon model. The hydrophobic and hydrophilic surfaces of the dimer are indicated in red and white, respectively. In (**C**), the relative locations of STG-1a, STG-1b, STG-2, AC0-12 (red), and myxothiazol (MYX: blue) are shown in overall and close-up views. In (**D**), the relative locations of UQ (orange), UQ_I_ (magenta), and UQ_II_ (pale orange) are shown in a close-up view. The structures of Ndi1-ubiquinone (PDB codes: 4GAV and 4G74) complexes are superposed to adapt this model^28,29^. In (**E**), the relative locations of STG-1a, STG-1b, STG-2, hexaethylene glycol (P6G: orange), triethylene glycol (PGE: pale orange), and Triton X-100 (TRT: purple) are shown in a close-up view. The structure of the substrate-free Ndi1 (PDB code: 4G6G) is superposed to adapt this model^29^.

Two stigmatellin molecules, STG-1 and STG-2 are bound to the hydrophobic surface of the membrane-anchor region of Ndi1 (Fig. 3A,B). STG-1 is located at the *si*-face of the adjacent FAD cofactor in the cavity formed by the C-terminal α15 helix and the β19-21 sheets of Ndi1 (Figs 1D and 3C). This cavity has been proposed to serve as a ubiquinone binding site based on the structures of the Ndi1-ubiquinone complex (PDB codes: 4GAV and 4G74) by our group^28^ and by Feng *et al*.^29^ (Fig. 3D).

**Figure 4:**
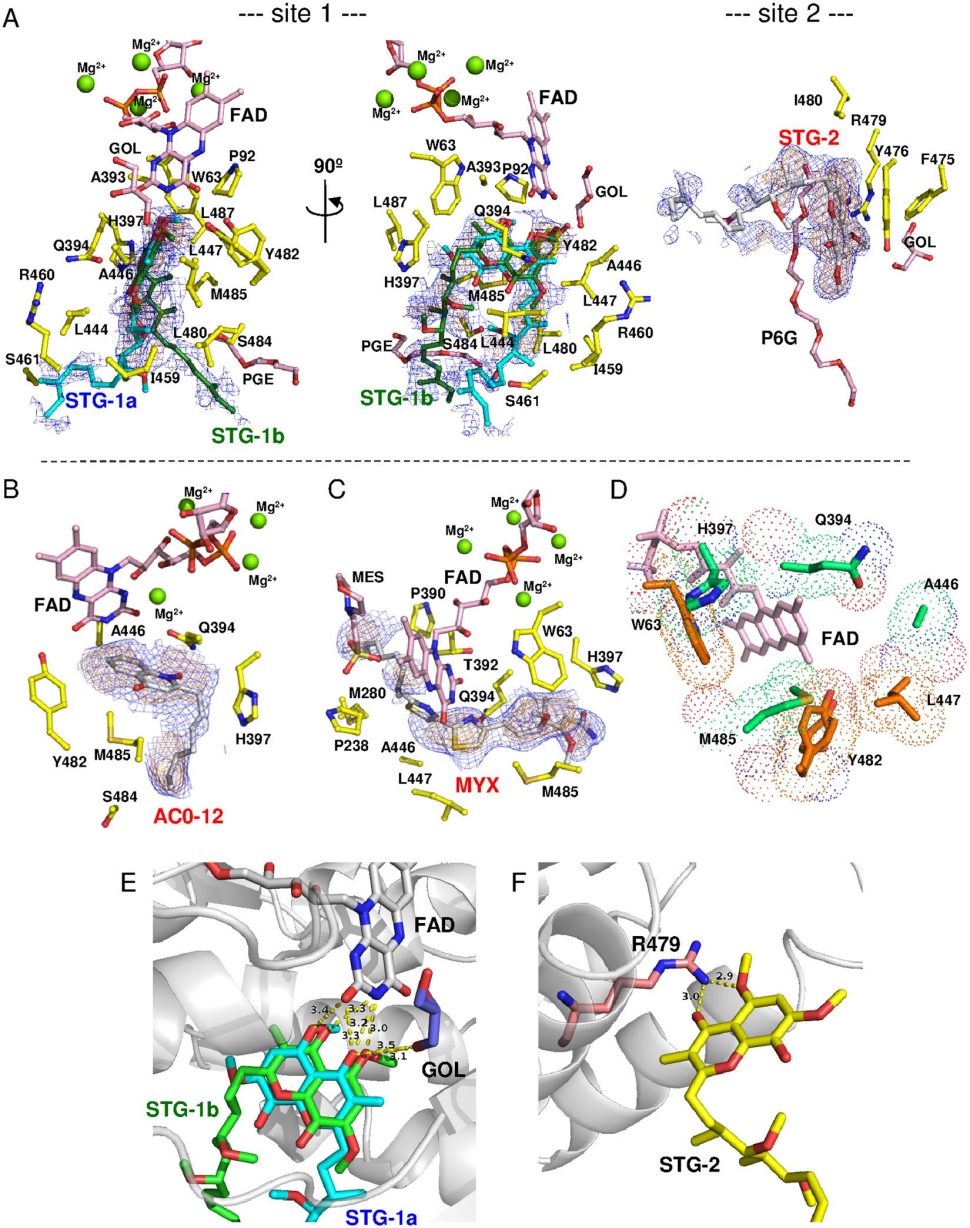
Amino acid residues contributing to inhibitor binding in Ndi1. (**A**–**C**) The residues interacting with stigmatellin at site 1 (STG-1a and STG-1b) and site 2 (STG-2) (**A**), AC0-12 (**B**), and myxothiazol (**C**) are represented by yellow sticks. The FAD cofactor, glycerol (GOL), triethylene glycol (PGE), hexaethylene glycol (P6G), and 2-morpholinoethanesulfonic acid (MES) are represented by pink sticks. The electron density maps calculated for inhibitors are represented by blue (1σ level) and orange (2σ level in **A** and **C**; 1.5σ level in **B**) nets. Both views are from the membrane-anchor side. (**D**) The residues interacting with two and three different inhibitors in the inhibitor-overlapping region are shown in orange and emerald green, respectively. (**E**,**F**) The residues contributing to the interaction with stigmatellin molecules by hydrogen bonding. The protein backbone is represented by a gray cartoon, with FAD (white), glycerol (GOL: blue), and Arg479 (R479: pale orange) molecules. (**E**) Interaction between the isoalloxazine ring of FAD and STG-1 (STG-1a: aqua; STG-1b: green). (**F**) Interaction between R479 and STG-2 (yellow).

**Figure 5:**
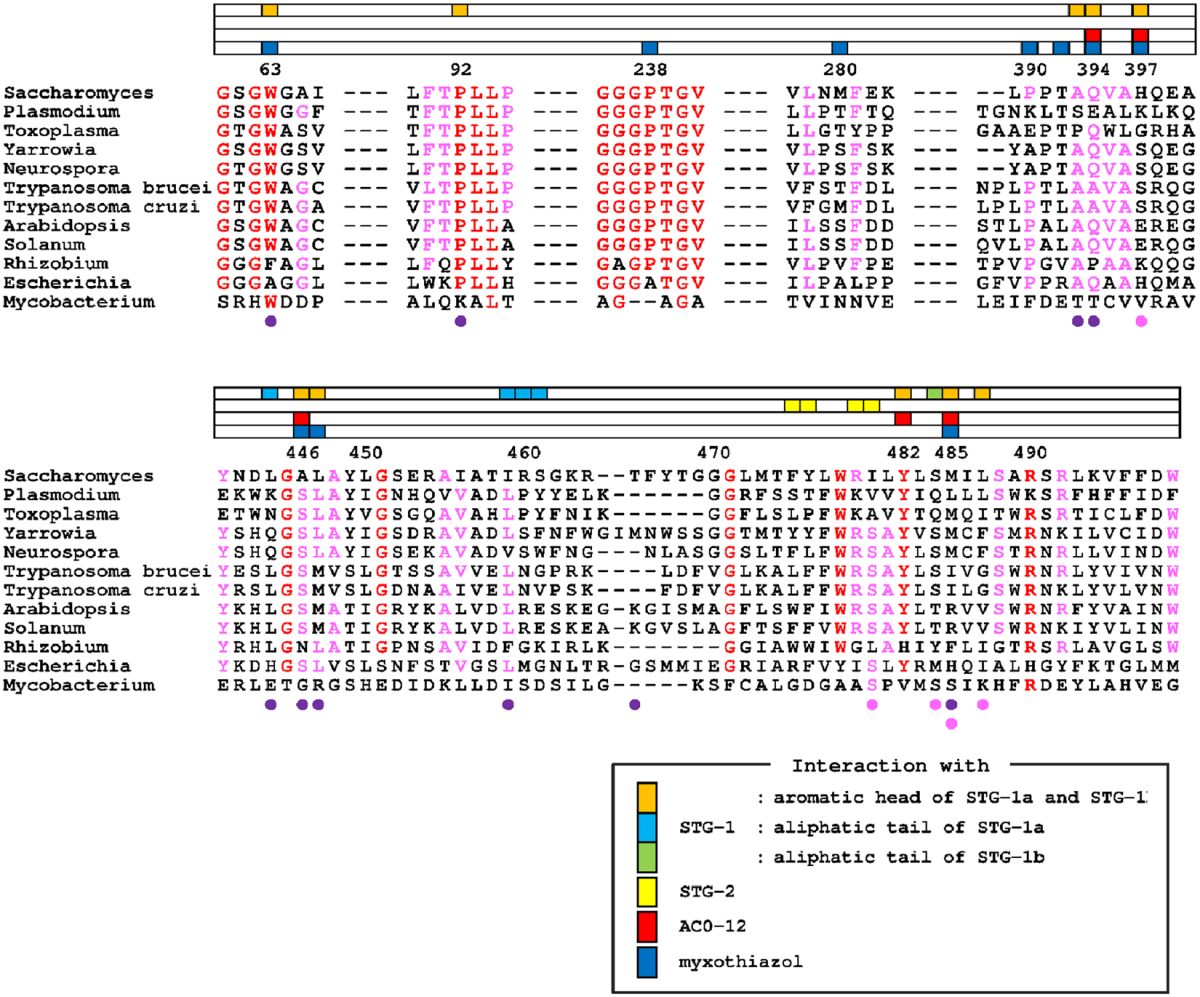
Multiple alignment of the amino acid sequences of NDH-2s. The amino acid residues interacting with the aromatic head groups of the STG-1a and STG-1b, the aliphatic tail of STG-1a, and the aliphatic tail of STG-1b are shown as orange, aqua, and green boxes, respectively. Those interacting with STG-2, AC0-12 and myxothiazol are shown as yellow, red, and blue boxes, respectively, in the lanes placed on the top of sequences. The amino acids residues interacting with ubiquinone at the UQ_I_ and UQ_II_ sites in the Ndi1-ubiquinone complex structures (PDB code: 4G74) are indicated by purple and pink circles at the bottom of the sequences, respectively^29^. The residues in red and pink indicate those that are conserved among 10-12, and 7-9 species, respectively. The alignment was produced from amino acid sequences from *Saccharomyces cerevisiae*, Ndi1 (CAA89160.1); *Plasmodium falciparum*, NDH-2 (XP_001352022.1); *Toxoplasma gondii*, NDH-2 (ABB17192.1); *Yarrowia lipolytica*, NDH-2 (CAA07265.1), *Neurospora crassa*, NDE-2 (EAA29772.1); *Trypanosoma brucei*, NDH-2 (XP_823167.1); *Trypanosoma cruzi*, NDH-2 (XP_812197.1); *Arabidopsis thaliana*, NDA1 (NP_563783.1); *Solanum tuberosum*, NDA1 (CAB52796.1); *Rhizobium etli*, NDH-2 (AAM54916.2); *Escherichia coli*, NDH-2 (CAA23586.1); and *Mycobacterium tuberculosis*, NDH-2 (KFZ73195.1).

The electron density of the STG-1 demonstrates that the inhibitor is bound to the enzyme in two distinct binding modes, STG-1a and STG-1b (site 1 in Fig. 4A). The aromatic head groups of the STG-1a and STG-1b overlap each other, while the aliphatic tails extend to different directions. The aromatic head groups of the STG-1a and STG-1b form hydrogen bonds with the isoalloxazine ring of the FAD cofactor (Fig. 4E) and are surrounded by W63, P92, A393, Q394, H397, A446, L447, Y482, M485, and L487 (site 1 in Figs 4A and 5). The aliphatic tail of STG-1a interacts with L444, I459, R460, and S461, whereas that of STG-1b interacts with S484 (site 1 in Fig. 4A). In contrast, STG-2 is bound to a groove formed by helixes α15 and α16 in the C terminal membrane-anchor domain of Ndi1 (Fig. 3A–C). The head group of the STG-2 is demonstrated by well-defined electron density, and it is lined by F475, Y476, R479, and I480 (site 2 in Fig. 4A) and formed the hydrogen bond with R479 (Fig. 4F), which is relatively conserved among different species (Fig. 5).

### AC0-12 and myxothiazol binding sites

In the structures of Ndi1-AC0-12 and Ndi1-myxothiazol complexes, AC0-12 and myxothiazol molecules are bound to a site that is identical to that of STG-1 (Fig. 3C). The hydrocarbon tail region (C8-C12) of AC0-12 in the Ndi1-AC0-12 complex has an extremely low electron density; thus, while the rest of the AC0-12 molecule can be observed, the hydrocarbon tail region cannot. The bound AC0-12 molecule is located in the vicinity of the *si*-face of the FAD isoalloxazine ring (Figs 1E and 4B) and forms hydrogen bonds with Q394 (Supplementary Fig. S1), which is relatively conserved across the amino acid sequences of various species (Fig. 5). The residues (Q394, H397, A446, Y482, and M485), which form the binding cavity of AC0-12 (Fig. 4B), are also involved in the binding site of the head group of the STG-1 (Figs 4D and 5). On the other hand, the electron density corresponding to myxothiazol extends from the *si*-face to the *re*-face of the FAD isoalloxazine ring (Figs 1F and 4C). The bithiazole group of myxothiazol is located adjacent to FAD and forms hydrogen bonds with the FAD isoalloxazine ring (Supplemental Fig. S1). This configuration is similar to that observed in the Ndi1-ubiquinone structure, which we described previously^28^ (Fig. 1A vs. 1F). The binding cavity of myxothiazol is constructed by 10 residues: W63, P238, M280, P390, T392, Q394, H397, A446, L447, and M485 (Fig. 4C), six of which (W63, Q394, H397, A446, L447, and M485) are also found among the residues lining the cavity for the head group of stigmatellin at the STG-1 site; four residues (P238, M280, P390, and T392) are specific to myxothiazol (Figs 4D and 5).

**Figure 6:**
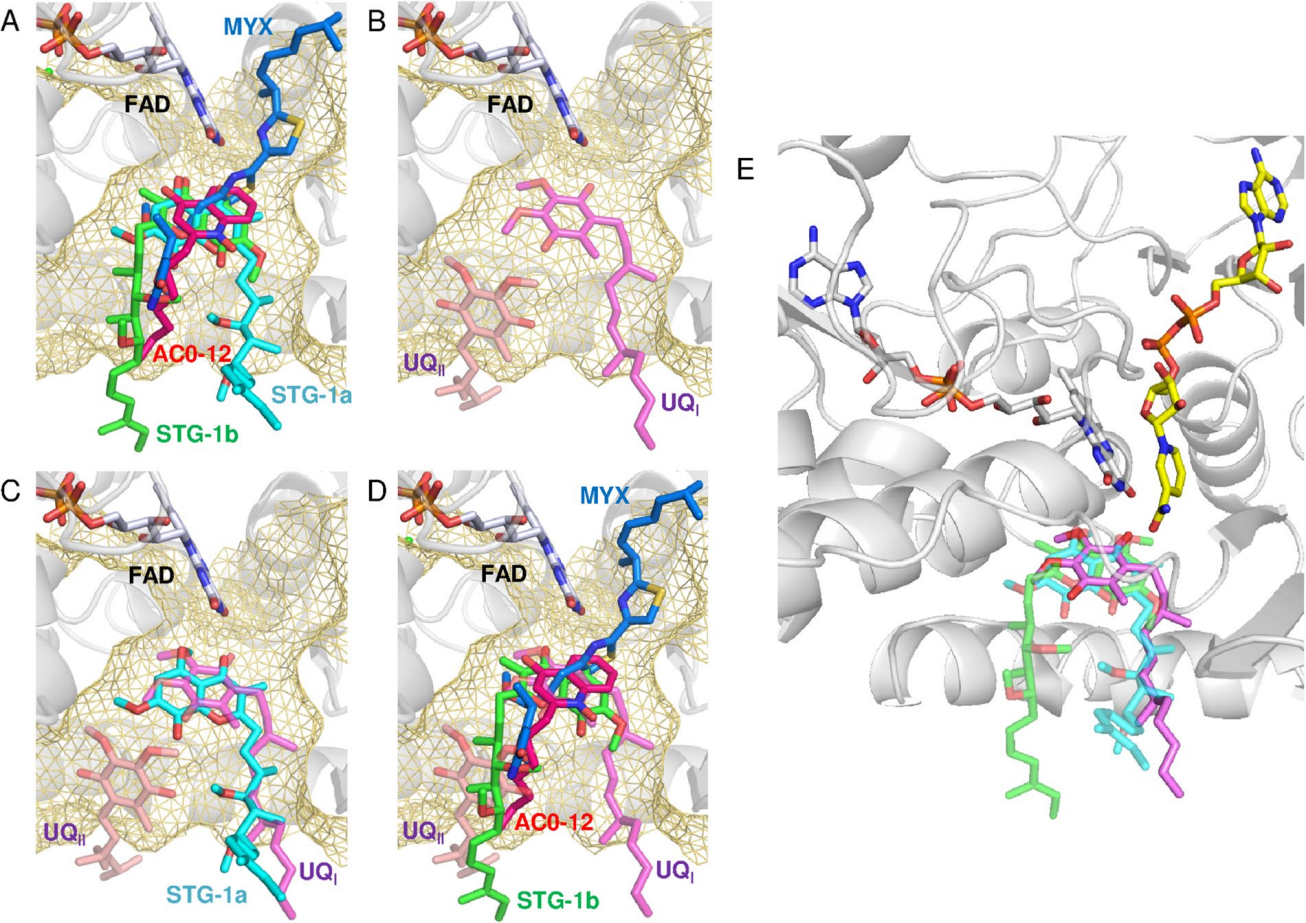
A comparison of the binding of stigmatellin, AC0-12 and myxothiazol to Ndi1, in relation to the UQ_I_ and UQ_II_ sites. (**A**) Close-up views of the overlap projection of the binding of stigmatellin (STG-1a: aqua; STG-1b: green), myxothiazol (blue) and AC0-12 (red) to Ndi1 (transparent gray). (**B**) A close-up view of the ubiquinone at the UQ_I_ (transparent pink) and UQ_II_ (transparent pale orange) sites, which were adapted by superimposing the Ndi1-ubiquinone complex structure (PDB code: 4G74) proposed by Feng *et al*.^29^. The amino acid residues that form a space for the UQ_I_ and UQ_II_ sites at the *si*-face of FAD are shown in transparent yellow meshes. (**C**,**D**) The overlap projection of ubiquinone in UQ_I_ and UQ_II_ sites with stigmatellin at STG-1a site (**C**) and stigmatellin at STG-1b, AC0-12 and myxothiazol (**D**). (**E**) The predicted UQ_I_ site in ubiquinone reduction by Ndi1. The structures determined for the Ndi1-stigmatellin, Ndi1-ubiquinone (PDB code: 4G74)^29^ and Ndi1-NAD^+^ complexes (PDB code: 4GAP)^28^ are superposed. The ubiquinone molecule (magenta) in the UQ_I_ site and NAD^+^ molecule (yellow) are located on the *si*-face and *re*-face of the isoalloxazine ring of FAD (white), respectively. The head group of stigmatellin in both positions of STG-1a (transparent aqua) and STG-1b (transparent green), as well as the alkenyl chain of stigmatellin in the position of STG-1a overlaps with the UQ_I_ site.

In summary, the aforementioned 10 residues that interact with the aromatic head group of STG-1a and STG-1b include the residues that interact with mixed-type inhibitors, AC-012 and myxothiazol, while 4 additional residues (P238, M280, P390, and T392) are specific for the interaction with myxothiazol. This large overlap reflects the observation that stigmatellin at the STG-1 site and the mixed-type inhibitors overlap near the FAD cofactor (Fig. 6A). In contrast, the residues that interact with the aliphatic tail of STG-1a and STG-1b and the residues that interact with STG-2 are specific for the interaction with stigmatellin.

### Kinetic analyses of the mutant enzymes

The comparative study on the modes of binding between stigmatellin at the STG-1a and STG-1b sites, AC0-12, and myxothiazol, allows us to recognize a unique structure of the aliphatic tail of stigmatellin at the STG-1a site (Fig. 6A). This unique structure appears to be related to the competitive nature of the inhibition of Ndi1 oxidoreductase activity by stigmatellin. It is therefore hypothesized that L444, I459, R460, and S461, which interact with stigmatellin at the STG-1a site, are also involved in the binding of the substrate ubiquinone (Fig. 5). This hypothesis was evaluated by examining the effects of mutations of the proximal L444 and I459 on the kinetics of the enzymatic reaction of Ndi1. The effect of the mutation of S484, which specifically interacts with stigmatellin at the STG-1b site, was examined as a control. Furthermore, the effects of mutations of W63, P92, A393, Q394, H397, L447, Y482, M485, and L487, which interact with the aromatic head group of stigmatellin at the STG-1a and STG-1b sites, were also examined. Moreover, to investigate the functional role of the STG-2 site in ubiquinone binding, a mutation was also introduced to R479. The wild-type and 22 mutated Ndi1 enzymes were expressed and purified as recombinant proteins, and their *K*_m_ and *V*_max_ values for the oxidoreductase reaction were determined using ubiquinone-1 as a substrate with NADH at a fixed concentration of 100 μM (Table 2).

**Table 2:** Kinetic parameters of the oxidoreductase activity of wild-type and mutant Ndi1 for ubiquinone-1.

	Interacting structure	*K*_m_ (μM)	*V*_max_ (μmol/min/mg)
Wild type	—	22.7 ± 1.0	1060 ± 6.1
L444D	aliphatic tail of STG-1a	37.6 ± 3.9	447 ± 6.8
L444N		23.9 ± 2.0	1333 ± 14
I459A		40.2 ± 4.1	2.90 ± 0.0042
I459N		30.3 ± 2.0	11.1 ± 0.096
I459W		75.6 ± 12	58.0 ± 1.5
W63F	aromatic head of STG-1a and STG-1b	53.1 ± 6.4	1450 ± 27
P92A		25.6 ± 2.2	11.3 ± 0.4
A393G		32.5 ± 1.9	882 ± 7.6
Q394G		30.8 ± 3.4	452 ± 8.1
Q394A		24.5 ± 1.7	391 ± 3.3
H397A		35.0 ± 2.7	463 ± 17
L447N		17.9 ± 1.8	140 ± 7.1
Y482F		23.3 ± 2.0	1090 ± 45
M485A		18.6 ± 1.1	292 ± 9.9
M485E		63.5 ± 7.8	1190 ± 96
L487A		16.4 ± 2.2	424 ± 29
S484F	aliphatic tail of STG-1b	24.5 ± 3.9	1160 ± 120
S484I		25.0 ± 1.7	1010 ± 33
R479I	STG-2	27.7 ± 1.2	0.313 ± 0.007
R479A		11.5 ± 0.58	316 ± 6.1
R479K		9.37 ± 0.48	305 ± 5.8
R479H		23.5 ± 1.4	1.06 ± 0.035

Among 5 mutants of L444 and I459, L444D, I459A, and I459W, increased the *K*_m_ value for ubiquinone-1 by 1.7-, 1.8-, and 3.3 fold, in comparison to the *K*_m_ value obtained with the wild-type enzyme, while I459A and I459N drastically reduced the *V*_max_ values (Table 2). I459N had a lower influence on the *K*_m_ value in comparison to I459A and I459W, in accordance with the smaller change in the amino acid size. L444N, a similar size-conservative mutation, has little effect on the *K*_m_ and *V*_max_ values.

Among 11 mutants of 9 residues interacting with the aromatic head group of stigmatellin, 5 mutants (W63F, A393G, Q394G, H397A, and M485E) increased the *K*_m_ values by 2.3-, 1.4-, 1.4-, 1.5-, and 2.8 fold, respectively (Table 2). The other 6 mutants (P92A, Q394A, L447N, Y482F, M485A, and L487A) had little effect on the *K*_m_ values (Table 2). However, the substantial reduction of the *V*_max_ values was observed with P92A (1% of the wild-type activity), L447N (13%), and M485A (28%) (Table 2). A partial decrease in the *V*_max_ value was observed with Q394G (43%), Q394A (37%), H397A (44%), and L487A (40%) (Table 2). Among them, Q394G and H397A also increased the *K*_m_ values as mentioned previously. In contrast, the significant decrease in the *V*_max_ value was not observed with W63F (137%), A393G (83%), and M485E (112%), which increased the *K*_m_ values (Table 2). Y482F, which had little effect on the *K*_m_ value, also had little effect on the *V*_max_ value (Table 2).

Two mutants of the residue interacting with stigmatellin at the STG-1b site—S484F and S484I—had no effect on either the *K*_m_ or *V*_max_ values. Four mutants of the residue interacting with stigmatellin at the STG-2 site—R479I (0.02%), R479A (30%), R479K (29%), and R479H (0.1%)—reduced the *V*_max_ values. R479A and R479K reduced the *K*_m_ values.

## Discussion

The present study identified four potent new inhibitors of yeast Ndi1: stigmatellin, AC0-12, myxothiazol, and ascochlorin. Stigmatellin, myxothiazol, and ascochlorin have novel carbon skeletons that are different from the known inhibitors of quinolone derivatives, including AC0-12. Among them, stigmatellin was determined to be the first competitive inhibitor against ubiquinone by the kinetic analysis of its effect on the Ndi1-mediated oxidoreductase reaction. The comparison of the structures of Ndi1 in complexes with stigmatellin, AC0-12 and myxothiazol revealed a unique binding mode of stigmatellin at the STG-1a site, with an aliphatic tail extending in a different direction from the other inhibitors. This unique binding of stigmatellin is suggested to be related to the competitive nature of its inhibition of Ndi1 oxidoreductase activity. Thus, it is hypothesized that the STG-1a site overlaps with the site of ubiquinone binding during the enzymatic reaction. In fact, the STG-1a site overlaps with the site of ubiquinone binding that was reported as the UQ_I_ site by Feng *et al*.^29^. Eight of the ten residues involved in the interaction with ubiquinone at UQ_I_ are shared by those involved in the interaction with stigmatellin at STG-1a (Fig. 5). The mutations of L444 and I459, which specifically interact with the aliphatic tail of stigmatellin at the STG-1a site, increased the *K*_m_ values for ubiquinone when mutations were non-conservative, and therefore caused significant changes in the size and charge of the amino acid, as was the case with L444D, I459W, and I459A. We propose that the STG-1a site represents the inherent ubiquinone binding site in yeast Ndi1.

The crystallographic analysis of the present study revealed three stigmatellin binding sites: STG-1a, STG-1b, and STG-2. STG-2 is not related to any of the reported binding sites of ubiquinone, NADH, or FAD^28,29^. The STG-2 site is more than 15 Å distant from the FAD binding site. Furthermore, the STG-2 site contained not only the stigmatellin molecule but also other hydrophobic molecules such as hexaethylene glycol in the cocrystal (P6G) (Fig. 3E and site 2 in Fig. 4A). Similarly, the detergent molecules (Triton X-100) were found in the same groove in the Ndi1 structure (PDB code: 4G6G) that was reported by Feng *et al*., (Fig. 3E)^29^. However, all of the mutations of R479, which specifically interact with stigmatellin at the STG-2 site, drastically reduced the *V*_max_ value, with no increase in the *K*_m_ value. Thus, we hypothesize that the binding of stigmatellin at the STG-2 site might be non-functional and an artificial observation.

In contrast, STG-1a apparently overlaps with the previously reported ubiquinone binding site, UQ_I_ (Fig. 6C), while STG-1b overlaps with UQ_II_ (Fig. 6D)^29^. The mutations of the amino acid residues, which interact with the aromatic head group that is common to both STG-1a and STG-1b, affected the enzymatic activity of Ndi1 by either increasing the *K*_m_ value or decreasing the *V*_max_ value (with the exception of Y482F). There was a reciprocal relationship between the effect on the *K*_m_ value and that on the *V*_max_ value: a greater increase in the *K*_m_ value was associated with a smaller reduction in the *V*_max_. Conversely, a greater decrease in the *V*_max_ value was associated with a smaller increase in the *K*_m_ value. M485E and W63F, which caused the greatest increase in the *K*_m_ value, caused no reduction (M485E)—or rather—an increase (W63F) in the *V*_max_ value. In contrast, P92A, L447N, and M485A, which caused the greatest decrease in the *V*_max_ value, caused no increase in the *K*_m_ value. These observations suggested that W63 and M485 are essential for ubiquinone binding and that P92, L447, and M485 might contribute to the Ndi1 activity. Since the aromatic head group of stigmatellin overlapped with the binding site of the mixed-type inhibitors, AC0-12 and myxothiazol, the amino acid residues interacting with the aromatic head group appear to be responsible for the mixed-type inhibition of the Ndi1 activity. On the other hand, the mutations of the residues specifically interacting with the aliphatic tail of stigmatellin at the STG-1a site increased the *K*_m_ value, when the mutation was non-conservative (L444D, I459W, and I459A). In contrast, the mutations of the residues specifically interacting with the aliphatic tail of stigmatellin at the STG-1b site had no effect on either the *K*_m_ or *V*_max_ values, despite the fact that the mutations were non-conservative (S484F, S484I). These observations suggest that the STG-1a site, especially the site of the aliphatic tail of stigmatellin—but not the STG-1b site—is responsible for the competitive inhibition of Ndi1 by stigmatellin. As a result, the UQ_I_ site, which overlaps with the STG-1a site (Fig. 6C)—but not the UQ_II_ site, which overlaps with the STG-1b site (Fig. 6D)—appears to represent the site of ubiquinone binding as a substrate during the enzymatic reaction.

As a result, 4 residues (W63, M485, L444, and I459) are suggested to contribute to the ubiquinone binding, because their non-conservative mutations consistently increased the *K*_m_ values. The aromatic character at W63 has been reported to be highly conserved among NDH-2s (Fig. 5) and suggested to stabilize the prosthetic group FAD in the enzyme^32^. The present study therefore identified a new role in substrate binding for these residues. Among the two residues (L444 and I459) that specifically interact with the aliphatic tail of STG-1a, the hydrophobicity at I459, but not L444, is conserved among the HDH-2s (Fig. 5).

The identification of ubiquinone binding as a substrate at the STG-1a/UQ_I_ site implies that the binding sites for two substrates—ubiquinone and NADH—are distinct. This configuration is apparently consistent with either (two-site) ping-pong or ternary complex mechanisms of the enzymatic reaction of Ndi1^23,25^. Our previous biochemical studies suggested that the binding sites for ubiquinone and NADH are distinct; thus, a model for the enzymatic reaction that was compatible with either two-site ping-pong or ternary complex mechanisms was proposed^25^. However, Feng *et al*., hypothesized that the binding of ubiquinone at the UQ_I_ site functioned as a structural component and not as a substrate^29,33^. Instead, they proposed that the substrate ubiquinone binds to the UQ_II_ site. In the present study, the mutations of the amino acid residue (S484) that specifically interacts with STG-1b, which overlaps with the UQ_II_ site, had no effect on the affinity for ubiquinone. The findings of the present study therefore support that the STG-1a/UQ_I_ site has a role as a site for substrate ubiquinone binding. STG-1b/UQ_II_ may not be functional in the enzymatic reaction of Ndi1. A comparison of the structure of the bacterial NDH-2^34,35^ to that of the yeast Ndi1 suggests that the side chain of R382 in bacterial NDH-2 may cause steric hindrance of the binding of quinone to the site corresponding to the UQ_II_ site in yeast Ndi1. This implies that there is only one quinone binding site in the bacterial NDH-2, which corresponds to the UQ_I_ site of yeast Ndi1, and that the site corresponding to the UQ_II_ site is non-functional in the bacterial NDH-2.

Our previous crystallographic study indicated that ubiquinone binds in close vicinity to FAD, penetrating the plane of the isoalloxazine ring of FAD with the isoprenoid side chain on the *si*-face and the quinone ring on the *re*-face (Fig. 1A)^28^. Since the quinone ring overlapped with NAD^+^, we discarded the possibility of the simultaneous binding of ubiquinone and NADH, and therefore propose that binding occurs via the one site ping-pong reaction mechanism. The binding pattern of ubiquinone that was observed in the previous study^28^ was similar to that observed with myxothiazol—one of the mixed-type inhibitors in the present study (Fig. 1F)—but was totally different from that observed at both the UQ_I_ and UQ_II_ sites in the crystallographic study by Feng *et al*., (Fig. 1C)^29^. On the other hand, STG-1a, to which the competitive inhibitor stigmatellin specifically binds, overlaps with the UQ_I_ site (Fig. 6C). Collectively, it is suggested that the ubiquinone binding site observed in our previous study may not represent the functional substrate binding site. The results of the present study are more consistent with those reported by Feng *et al*., which are compatible with either two-site ping-pong or ternary complex mechanisms. Ubiquinone-2 was used for co-crystallization in our previous study, while ubiquinone-4, which is more hydrophobic than ubiquinone-2 and more relevant to the ubiquinone that exits in the yeast cells, was used in the study by Feng *et al*.^29^. The difference in the hydrophobicity of ubiquinone derivatives might have caused the difference in the identification of the ubiquinone binding sites in these two studies.

In conclusion, the present study identified the first competitive inhibitor of the yeast Ndi1. The cocrystal structure of Ndi1 in complex with this competitive inhibitor and the biochemical studies with the mutants of Ndi1 in the relevant amino acid residues revealed the binding site for the inhibitor, which provides the structural basis for the competitive nature of its inhibition of Ndi1. This STG-1a site, a competitive binding site for the inhibitor, is therefore suggested to represent the binding site for the substrate ubiquinone (Fig. 6E). This configuration of the ubiquinone binding is consistent with the hypothesis that the Ndi1-mediated oxidoreductase reaction occurs via either a two-site ping-pong mechanism or a ternary complex mechanism.

## Material and Methods

### Materials

Ubiquinone-1, stigmatellin, myxothiazol, and ascochlorin were purchased from Sigma-Aldrich (St. Louis, MO). AC0-12 was synthesized according to the method of 1-hydroxy-2-alkyl-4(1 H) quinolone synthesis^36^. The MagicMedia™ *E. coli* Expression Medium was from Life Technologies (Carlsbad, CA). The Ultra Yield Flask™ was from Thomson Instrument Company (Oceanside, CA). The HisTrap HP column was from GE Healthcare (Buckinghamshire, England). The Econo-Pac 10DG column was from Bio-Rad (Hercules, CA). All of the other chemicals were of reagent grade and were obtained from commercial sources.

### Purification

The *Saccharomyces cerevisiae* Ndi1 enzyme was essentially purified and crystallized according to a previously described method^25,28^. Detailed methods of the expression and purification of Ndi1 can be found as Supplementary Methods.

### Site-directed mutagenesis

A detailed methods of the amino acid replacements of Ndi1 can be found as Supplementary Methods.

### Enzyme assays and the data analysis

NADH-ubiquinone oxidoreductase activity was measured as described previously^23^. In brief, the reaction was started by adding 100 µM NADH to the enzyme mixture containing 10 ng/mL Ndi1, 50 mM sodium phosphate (pH 6.0) and 1 mM EDTA, which was pre-incubated with various concentrations of ubiquinone-1 for 1 min. The decrease in 340 nm absorbance was measured as an indication of the oxidation of NADH (ε = 6.22 mM^−1^ cm^−1^) with a Shimadzu UV2000 spectrophotometer (Kyoto, Japan). The enzymatic activity obtained with 100 µM NADH and 60 µM ubiquinone-1 in the absence of any inhibitors (100% activity) catalyzed the oxidation of 1,890 μmol NADH/min/mg Ndi1. The concentrations of inhibitors required to obtain the half-maximal inhibition (IC_50_) were estimated by non-linear regression dose-response curves. The inhibition mode of each inhibitor for ubiquinone-1 was determined by the intersection point in double-reciprocal plots (Fig. 2B–E). To objectively evaluate the inhibition mode, the data of the inhibition kinetics were fitted to the Eq. (1) of mixed model inhibition in the Prism6 software program (GraphPad Software, Inc., La Jolla, CA).

1 $${\rm{V}}=\frac{{{\rm{V}}}_{{\max }}[S]}{[S](1+\frac{\,[I]}{\alpha {K}_{i}})+{K}_{m}(1+\frac{[I]}{{K}_{i}})}$$

This equation provides two dissociation constants, one for the binary enzyme-inhibitor complex (*Ki*), the other for the ternary enzyme-substrate-inhibitor complex (α*Ki*). The value of α ranges from 0 to infinity, and determines the inhibition mechanism. When the constant α shows unity, the inhibition mechanism is considered to be noncompetitive; when α is closer to infinity, the inhibition mechanism is considered to be competitive; when α is closer to 0, the inhibition mechanism is considered to be uncompetitive.

To determine the kinetic parameters of wild-type and mutant enzymes for ubiquinone-1, the NADH oxidation rates were measured in a fixed concentration of NADH (100 µM) and different concentrations of ubiquinone-1 (2.4–60 µM). Apparent *K*_m_ and *V*_max_ values were obtained by fitting a data set of initial velocity versus substrate concentration to the Michaelis-Menten model equation in the Prism6 software program.

### Crystallization

The purified *Saccharomyces cerevisiae* Ndi1 enzyme was essentially crystallized according to a previously described method^28^ by the hanging-drop vapor diffusion method. Crystals of the Ndi1-stigmatellin, Ndi1-AC0-12, and Ndi1-myxothiazol complexes were prepared by co-crystallization in the presence of 1 mM inhibitors. The protein solution (10 mg/ml, 20 mM Mops-KOH pH 7.0, 5% (*v*/*v*) glycerol, 0.02% (*w*/*v*) DDM, 1 mM inhibitor) was mixed with an equal volume of the reservoir solution (50 mM Mes-NaOH pH 6.2, 43% (*v*/*v*) PEG400, 100 mM NaCl, 2% (*v*/*v*) ethylene glycol, 5% (*v*/*v*) glycerol) and incubated at 20 °C. The crystals reached maximum dimensions of 0.3 × 0.2 × 0.05 mm^3^ in complex with AC0-12 and in complex with myxothiazol; this size was almost the same as that of the previously reported crystals^28^. The crystals in complex with stigmatellin were thin triangles with maximum dimensions (one side) of 0.3 mm and a thickness 0.05 mm.

### Data collection and phasing

A crystal was scooped up from a hanging droplets using a cryo-loop and was flash-frozen in liquid nitrogen gas. The X-ray diffraction data of the Ndi1-stigmatellin and Ndi1-AC0-12 complex crystals were collected at −173 °C on beamline BL44XU at SPring-8 (Harima, Japan) and that of the Ndi1-myxothiazol crystal was collected at −173 °C on beamline BL17A at Photon Factory, the High Energy Accelerator Organization (Tsukuba, Japan). All of the diffraction data sets were processed and scaled with the HKL2000 program package^37^, as shown in Table S1. The determination of the structure was carried out by molecular replacement with Molrep in the CCP4 software program^38^ using the atomic model of the ligand-free Ndi1 structure (PDB code: 4G9K) as a search model.

### Refinement

The model structures of Ndi1-stigmatellin, Ndi1-AC0-12 and Ndi1-myxothiazol were refined using Refmac5 in the CCP4 software program^39^ at resolutions of 1.85, 3.4 and 3.2 Å, respectively. Further refinement and model rebuilding were performed using Refmac5 and COOT^40^. The crystal of Ndi1-stigmatellin contains one Ndi1 and two bound stigmatellin molecules in the asymmetric unit. Crystals of Ndi1-AC0-12 and -myxothiazol complexes contain one dimer molecule in each of the asymmetric units. All structures were refined to final *R*_work_/*R*_free_ values of 0.158/0.192 (Ndi1-stigmatellin), 0.207/0.299 (Ndi1-AC0-12), and 0.220/0.285 (Ndi1-myxothiazol), respectively. The results of the structure determination are summarized in Table S1. The figures showing the protein structures were prepared with the PyMol software program (http://www.pymol.org/).

### Data Availability

The atomic coordinates and structural factors have been deposited in the Protein Data Bank, www.pdb.org (Ndi1-stigmatellin, 5YJW; Ndi1-AC0-12, 5YJY; and Ndi1-myxothiazol, 5YJX).

## Electronic supplementary material

Supplementary Information
