# Supplementary material for: Ubiquinone binding site of yeast NADH dehydrogenase revealed by structures binding novel competitive- and mixed-type inhibitors
Source: Sci Rep. 2018 Feb 5;8:2427. doi: 10.1038/s41598-018-20775-6 (PMC5799168; doi:10.1038/s41598-018-20775-6)
Supplement: Supplementary file 1 — Supplementary Information [file 41598_2018_20775_MOESM1_ESM.pdf]

Supplementary Information

Ubiquinone binding site of yeast NADH dehydrogenase revealed by structures binding novel competitive- and mixed-type inhibitors.

Tetsuo Yamashita[1,*,†], Daniel Ken Inaoka[2,3,†], Tomoo Shiba[4,†], Takumi Oohashi[2], So Iwata[5,6,7,8,9], Takao Yagi[10], Hiroaki Kosaka[1,‡], Hideto Miyoshi[11], Shigeharu Harada[4,*], Kiyoshi Kita[2,3], Katsuya Hirano[1]

[1]Department of Cardiovascular Physiology, Faculty of Medicine, Kagawa University, Kita-gun, Kagawa 761-0793, Japan.

[2]Department of Biomedical Chemistry, Graduate School of Medicine, The University of Tokyo, Tokyo 113-0033, Japan

[3]School of Tropical Medicine and Global Health, Nagasaki University, 1-12-4, Sakamoto, Nagasaki 852-8523, Japan

[4]Department of Applied Biology, Graduate School of Science and Technology, Kyoto Institute of Technology, Kyoto 606-8585, Japan

[5]Division of Molecular Biosciences, Membrane Protein Crystallography Group, Imperial College, London SW7 2AZ, UK.

[6]Membrane Protein Laboratory, Diamond Light Source, Harwell Science and Innovation Campus, Chilton, Didcot, Oxfordshire OX11 0DE, UK.

[7]Japan Science and Technology Agency, Exploratory Research for Advanced Technology, Human Receptor Crystallography Project, Yoshida-Konoe-cho, Sakyo-ku, Kyoto 606-8501, Japan.

[8]Department of Cell Biology, Graduate School of Medicine, Kyoto University, Yoshida-Konoe-cho, Sakyo-Ku, Kyoto 606-8501, Japan.

[9]Systems and Structural Biology Centre, RIKEN, 1-7-22 Suehiro-cho Tsurumi-ku, Yokohama, Kanagawa 230-0045, Japan.

[10]Department of Molecular and Experimental Medicine, The Scripps Research Institute, La Jolla, California 92037, USA.

[11]Division of Applied Life Sciences, Graduate School of Agriculture, Kyoto University, Sakyo-ku, Kyoto 606-8502, Japan.

*Correspondence: harada@kit.ac.jp (S.H.)
tyama@med.kagawa-u.ac.jp (T. Yamashita)

†These authors contributed equally to this work.

‡Present address: Osaka Jikei College, 1-2-8 Miyahara, Yodogawa-Ku, Osaka 532-0003, Japan.

Supplementary Methods

The expression and purification of Ndi1. The mature Ndi1, which lacks a mitochondrial targeting signal, is expressed as an recombinant protein tagged with $(His)_{10}$ by pET16b vector and *E. coli* BL21(DE3)pLysS. The construction of the expression plasmid, pET16b(NDI1-m), was described previously [1]. The bacterial cells harboring pET16b(NDI1-m) were grown in 5 mL of 2 × YT medium containing 100 μg/mL of ampicillin and 20 μg/mL of chloramphenicol for about 7–8 h with rotation at 220 rpm at 37°C and then they were inoculated to 500 mL of MagicMedia™ *E. coli* expression medium (Life Technologies) containing 100 μg/mL of ampicillin for the expression of recombinant proteins. This media does not require isopropyl β-D-thiogalactopyranoside for the induction of the expression. The cells were cultured in highly aerated conditions using a 2.5 L baffled flask (Ultra Yield flask™) with rotation at 300 rpm at 37°C. After 15-16 h, the cells were collected by centrifugation at 8,000 × *g* for 10 min.

The wild-type Ndi1 used in the crystallization trial and the evaluation experiments of inhibitors was purified using a detergent exchange procedure, as described previously [1]. The harvested cells were homogenized in 50 mM Tris-HCl (pH 8.0 at 25°C), 1 mM EDTA, and protease inhibitor cocktail (cOmplete ULTRA Tablets; Roche) by freeze-and-thaw cycles (repeated two times) in liquid nitrogen with sonication for 3 min (Output: 20%, 1/4” microchip, SONIFIER 250D-Advanced; Branson). The homogenates were centrifuged at 10,000 × *g* for 10 min at 4°C to remove unbroken cells and inclusion bodies, and the supernatant was further centrifuged at 183,000 × *g* for 90 min at 4°C. The pellet (membrane fraction) was then suspended at 5 mg of protein/ml in 50 mM Tris-HCl (pH 8.0 at 4°C), 200 mM NaCl, 0.1 mM EDTA, 1 mM phenylmethylsulfonyl fluoride, and 10% glycerol (buffer A). After adding Triton X-100 at a final concentration of 0.3% (*w*/*v*), the sample was incubated for 30 min at 4°C with slow stirring and centrifuged at 183,000 × *g* for 90 min at 4°C. The supernatant was loaded onto a Ni-sepharose column (HisTrap HP, 5 mL; GE healthcare), which was equilibrated with buffer A containing 0.02% (*w*/*v*) Triton X-100. The column was washed with 10 column volumes of buffer A containing 0.1% (*w*/*v*) Triton X-100 and 15 mM histidine. The column was subjected to detergent exchange by washing with 5 column volumes of buffer A, 10 column volumes of buffer A containing 0.1% (*w*/*v*) dodecyl-β-D-maltoside (DDM), and 5 volumes of buffer A containing 0.02% (*w*/*v*) DDM. Thereafter, the enzyme was eluted with buffer A containing 0.02% (*w*/*v*) DDM and 200 mM histidine and 50 mM imidazole. The eluents were immediately loaded onto a desalting column (Econo-Pac 10DG, 10 ml; Bio-Rad), which had been equilibrated with 50 mM Mops-NaOH (pH 7.0) containing 0.1 mM EDTA, 10% (*w*/*v*) glycerol, and 0.02% (*w*/*v*) DDM. The desalted specimen was then concentrated with Amicon-Ultra (molecular weight cut-off 30K; Millipore) to 10 mg of protein/ml. The yield of Ndi1 contains >20 mg/L of culture media. The purity of the Ndi1 preparations was approximately 90%, as determined by the intensity of the 57 kDa band on a SDS-PAGE gel stained with CBB Stain One Super (Nacalai, Japan). The purified recombinant Ndi1 was quickly frozen in liquid nitrogen and stored at −80°C until use.

Site-directed mutagenesis. The amino acid replacements of Ndi1 were basically performed with PrimeSTAR mutagenesis basal kit (Takara, Japan) using pET16b(NDI1-m) (2) and a synthetic oligonucleotide primer set (Table S2). The mutagenesis of H397 was performed with a QuikChange II XL site-directed mutagenesis kit (Stratagene, CA). Each mutation was confirmed through DNA sequencing (DNA sequencing service, Eurofins). The purification procedure of wild-type and mutant enzymes, used for the determination of the kinetic parameters (Table 2), was basically the same as the above procedure, without the replacement of the detergent from Triton X-100 with DDM. After washing the enzyme with buffer A containing 0.1% Triton X-100 and 15 mM histidine, 0.02% Triton X-100 was added to all buffer as a substitute for DDM. With the exception of 5 mutants containing L444D, I459A, I459N, R479I, and R479H, which showed a yield <10% of wild-type (1.04, 1.86, 1.58, 1.06, and 1.74 mg of protein/L of culture media, respectively), the yields of the mutant enzymes were comparable to the yield of the wild-type enzyme.

References

1 Yamashita, T., Nakamaru-Ogiso, E., Miyoshi, H., Matsuno-Yagi, A. & Yagi, T. Roles of bound quinone in the single subunit NADH-quinone oxidoreductase (Ndi1) from Saccharomyces cerevisiae. *J Biol Chem* **282**, 6012-6020, (2007).

Supplementary Figures

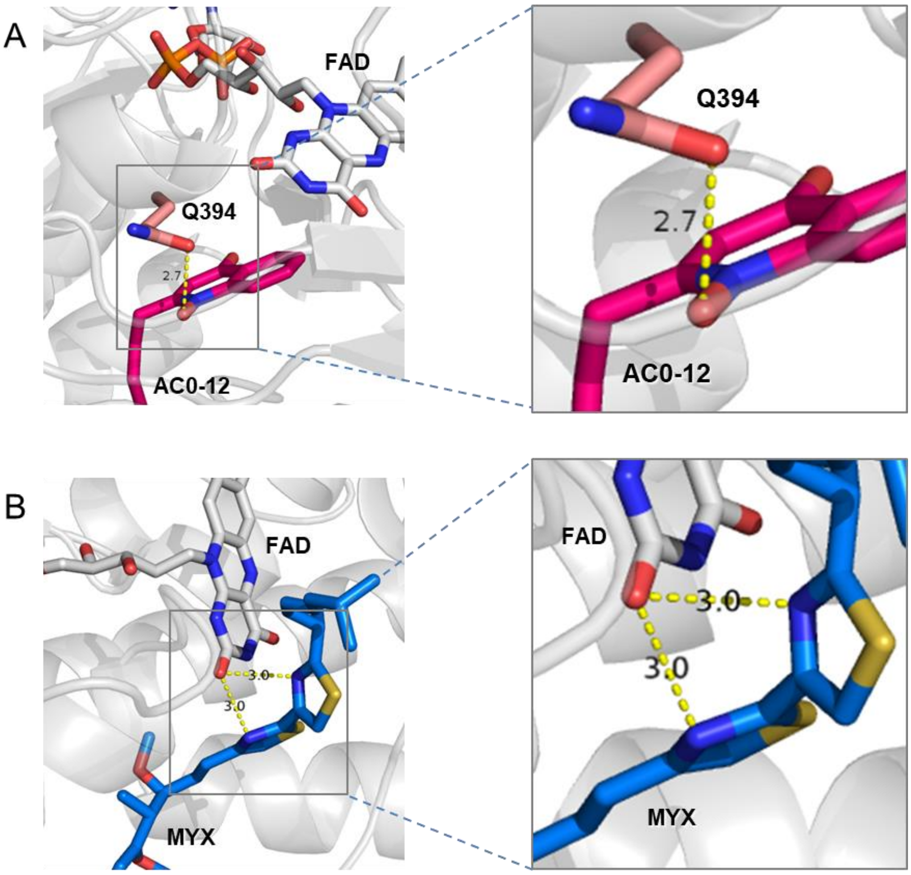

Figure S1. Amino acid residues contributing to the interaction with AC0-12 and myxothiazol molecules by hydrogen bonding. The protein backbone is represented by a gray cartoon, and the FAD and Gln394 (Q394) molecules are indicated with white and pale orange, respectively. (*A*) Interaction between Q394 and AC0-12 (red). (*B*) Interaction between the isoalloxazine ring of FAD and myxothiazol (blue).

Table S1. Data collection and refinement statistics

Data set	NDH2-stigmatellin	NDH2-AC0-12	NDH2-myxothiazol
**Data collection**			
Space group	$C222_1$	$P2_12_12_1$	$P2_12_12_1$
Cell parameters			
a / b / c (Å)	116.7, 128.5, 86.7	69.9, 115.8, 166.5	70.3, 116.4, 166.6
X-ray source	SPring8 BL44XU	SPring8 BL44XU	KEK-PF 17A
Wavelength (Å)	0.9000	0.9000	0.9800
Temperature (K)	100	100	100
Resolution (Å)	50.0-1.85 (1.88-1.85)	50.0-3.4 (3.46-3.4)	50-3.2 (3.26-3.2)
Total reflections	301,479	90,367	147,138
Unique reflections	55,879	18,371	22,984
Completeness (%)	99.9 (100.0)	97.3 (98.6)	98.1 (99.6)
R_{merge} I	0.086 (0.786)	0.094(0.764)	0.125 (0.609)
$I/\sigma(I)$	7.1 (1.8)	7.9 (1.2)	7.1 (2.7)
Redundancy	5.4 (5.0)	4.9 (4.6)	6.5 (5.8)
**Refinement**			
Resolution (Å)	30 – 1.85	30 – 3.4	20 – 3.2
No. of reflections	49,008	15,012	19,430
R_{work} / R_{free}	0.182 / 0.204	0.207 / 0.299	0.220 / 0.285
Average B-factors (Å^2)	24.3	62.9	78.4
rmsd from ideal values			
Bond length (Å)	0.007	0.011	0.008
Bond angle (°)	1.44	1.61	1.43
Residues in the Ramachandran plot (%)*	92.1	79.5	82.3
Most favored regions	7.9	19.1	17.0
Additionally allowed regions	0.0	1.4	0.6
Generously allowed regions	0.0	0.0	0.0
Disallowed regions			
Protein Data Bank ID code	5YJW	5YJY	5YJX

* calculated by Procheck

Table S2. The primers used for the site-directed mutagenesis of Ndi1

mutant	forward primer	reverse primer
W63F	5'-TCGGGGT**TC**GGAGCTATTTCGTTTTTAAAGCAC-3'	5'-AGCTCC**GA**ACCCCGAACCCAGTATCAGCACGTT-3'
A393G	5'-CCAACCG**G**CCAAGTAGCGCACCAAGAG-3'	5'-TACTTGG**C**CGGTTGGTGGCAACCCAGC-3'
Q394G	5'-ACCGCC**GG**AGTAGCGCACCAAGAGGCC-3'	5'-CGCTACT**CC**GGCGGTTGGTGGCAACCC-3'
Q394A	5'-ACCGCC**GC**AGTAGCGCACCAAGAGGCCGAATAT-3'	5'-CGCTACT**GC**GGCGGTTGGTGGCAACCCAGCAAA-3'
H397A	5'-GCCCAAGTAGCG**GCC**CAAGAGGCCG-3'	5'-CGGCCTCTTG**GGC**CGCTACTTGGGC-3'
L444D	5'-AACGAT**GAT**GGTGCCTTAGCATACCTGGGATCC-3'	5'-GGCACC**ATC**ATCGTTGTATTTGAAAGGTTTAAA-3'
L444N	5'-AACGAT**AAT**GGTGCCTTAGCATACCTGGGATCC-3'	5'-GGCACC**ATT**ATCGTTGTATTTGAAAGGTTTAAA-3'
L447N	5'-GGTGCC**AAT**GCATACCTGGGATCCGAAAGGGCC-3'	5'-GTATGC**ATT**GGCACCTAAATCGTTGTATTTGAA-3'
I459A	5'-GCAACC**GC**ACGTTCCGGTAAGAGAACATTTTAC-3'	5'-GGAACGT**GC**GGTTGCAATGGCCCTTTCGGATCC-3'
I459N	5'-GCAACCA**AT**CGTTCCGGTAAGAGAACATTTTAC-3'	5'-GGAACG**AT**TGGTTGCAATGGCCCTTTCGGATCC-3'
I459W	5'-GCAACC**TGG**CGTTCCGGTAAGAGAACA-3'	5'-GGAACG**CCA**GGTTGCAATGGCCCTTTC-3'
R479I	5'-CTTATTCA**T**AATTTTGTACTTGTCCATGATTCT-3'	5'-GTACAAAATT**A**TCCATAAGTAGAAGGTCATTAAGC-3'
R479A	5'-TTATGG**GC**AATTTTGTACTTGTCCATG-3'	5'-CAAAATT**GC**CCATAAGTAGAAGGTCAT-3'
R479K	5'-TTATGGA**AG**ATTTTGTACTTGTCCATG-3'	5'-CAAAAT**CT**TCCATAAGTAGAAGGTCAT-3'
R479H	5'-TTATGG**CAC**ATTTTGTACTTGTCCATG-3'	5'-CAAAAT**GTG**CCATAAGTAGAAGGTCAT-3'
Y482F	5'-ATTTTGT**T**CTTGTCCATGATTCTATCT-3'	5'-GGACAAG**A**ACAAAATTCTCCATAAGTA-3'
S484F	5'-TACTTG**TT**CATGATTCTATCTGCAAGA-3'	5'-AATCATG**AA**CAAGTACAAAATTCTCCA-3'
S484I	5'-TACTTG**AT**CATGATTCTATCTGCAAGA-3'	5'-AATCATG**AT**CAAGTACAAAATTCTCCA-3'
M485A	5'-TTGTCC**GC**GATTCTATCTGCAAGATCG-3'	5'-TAGAATC**GC**GGACAAGTACAAAATTCT-3'
M485E	5'-TTGTCC**GA**GATTCTATCTGCAAGATCG-3'	5'-TAGAATC**TC**GGACAAGTACAAAATTCTCC-3'
L487A	5'-ATGATT**GC**ATCTGCAAGATCGAGATTA-3'	5'-TGCAGAT**GC**AATCATGGACAAGTACAA-3'

Bold typefaces indicate the introduced mutation sites.
